# Exploring the Use of Helicogenic Amino Acids for Optimising Single Chain Relaxin-3 Peptide Agonists

**DOI:** 10.3390/biomedicines8100415

**Published:** 2020-10-14

**Authors:** Han Siean Lee, Shu Hui Wang, James T. Daniel, Mohammed Akhter Hossain, Richard J. Clark, Ross A. D. Bathgate, K. Johan Rosengren

**Affiliations:** 1 School of Biomedical Sciences, Faculty of Medicine, The University of Queensland, Brisbane, QLD 4072, Australia; hslee@uqconnect.edu.au (H.S.L.); shu.wang2@uq.net.au (S.H.W.); james.daniel@uqconnect.edu.au (J.T.D.); richard.clark@uq.edu.au (R.J.C.); 2 The Florey Institute of Neuroscience and Mental Health, The University of Melbourne, Melbourne, VIC 3052, Australia; akhter.hossain@unimelb.edu.au (M.A.H.); bathgate@unimelb.edu.au (R.A.D.B.); 3 School of Chemistry, The University of Melbourne, Melbourne, VIC 3010, Australia; 4 Department of Biochemistry and Molecular Biology, The University of Melbourne, Melbourne, VIC 3010, Australia

**Keywords:** relaxin-3, RXFP3, α-aminoisobutyric acid, helical stapling

## Abstract

Relaxin-3 is a highly conserved two-chain neuropeptide that acts through its endogenous receptor the Relaxin Family Peptide-3 (RXFP3) receptor. The ligand/receptor system is known to modulate several physiological processes, with changes in food intake and anxiety-levels the most well studied in rodent models. Agonist and antagonist analogues based on the native two-chain peptide are costly to synthesise and not ideal drug leads. Since RXFP3 interacting residues are found in the relaxin B-chain only, this has been the focus of analogue development. The B-chain is unstructured without the A-chain support, but in single-chain variants structure can be induced by dicarba-based helical stapling strategies. Here we investigated whether alternative helical inducing strategies also can enhance structure and activity at RXFP3. Combinations of the helix inducing α-aminoisobutyric acid (Aib) were incorporated into the sequence of the relaxin-3 B-chain. Aib residues at positions 13, 17 and 18 partially reintroduce helicity and activity of the relaxin-3 B-chain, but other positions are generally not suited for modifications. We identify Thr21 as a putative new receptor contact residue important for RXFP3 binding. Cysteine residues were also incorporated into the sequence and cross-linked with dichloroacetone or α, α’-dibromo-*m*-xylene. However, in contrast to previously reported dicarba variants, neither were found to promote structure and RXFP3 activity.

## 1. Introduction

Relaxin-3 is a neuropeptide predominantly expressed in neurons of the nucleus incertus, which project widely to forebrain regions such as the hypothalamus, hippocampus and septum [1], and overlap regions expressing its cognate receptor, the Relaxin Family Peptide 3 (RXFP3) Receptor [2]. Relaxin-3 is highly conserved [3], and has been shown to play an important role in several neurophysiological processes. Using animal models, relaxin-3 has been shown to increase food intake in satiated rats [4,5,6] and reduce stress [7,8,9], whereas antagonist treatment can reduce food consumption in rodents and also mitigate stress-induced alcohol seeking behaviour [10,11,12]. The physiological significance of relaxin-3/RXFP3 signalling has been recently reviewed [13]. As relaxin-3 has potential as a therapeutic agent in combating obesity and psychological disorders, a lot of focus has been given to its structure–activity relationships (SAR) to develop peptide analogues as potential drug leads. 

**Figure 1:**
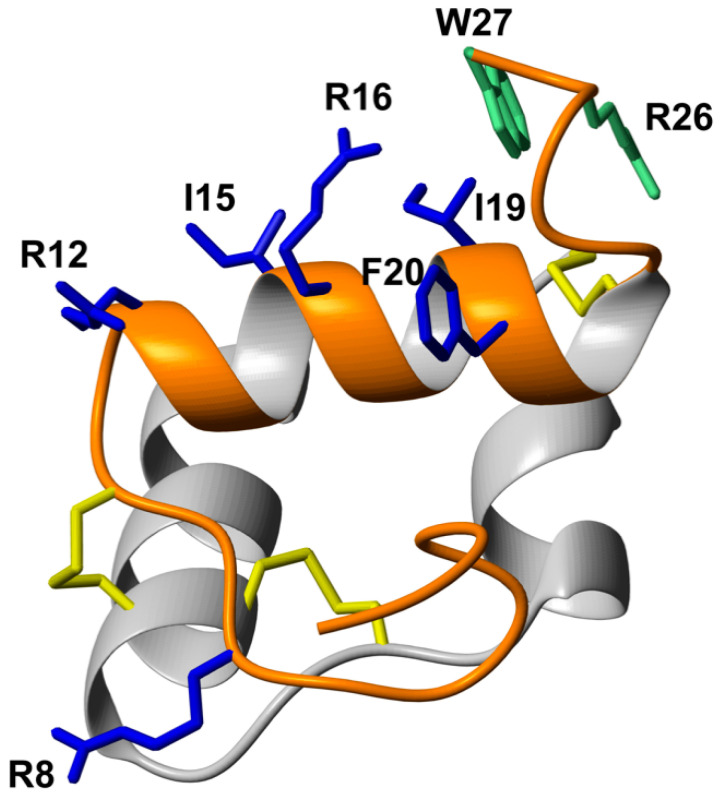
Three-dimensional structure of relaxin-3. The A-chain and B-chain are shown in grey and dark orange respectively. The structure is braced by an intra-chain disulfide in the A-chain and two inter-chain disulfide bonds, shown in yellow. Residues in blue are important for binding to RXFP3, whereas the green residues are required to activate the receptor.

A member of the human insulin/relaxin superfamily of peptide hormones, relaxin-3 (H3 relaxin) consists of an A-chain with a helix-turn-helix motif and a B-chain with a single helix, connected by two interchain disulfide bonds and one intrachain disulfide bond in the A-chain (Figure 1) [14]. Relaxin-3 binds and signals through its class A G protein-coupled receptor (GPCR), RXFP3 [15]. However, the peptide also has high affinity for receptors of the related peptides relaxin-2 and insulin-like peptide 5 (INSL5), RXFP1 and RXFP4, respectively [16,17]. Several studies have addressed this selectivity issue in order to develop new analogues for studying RXFP3 signalling in vivo. The potency at RXFP3 is retained but the interaction with RXFP1 is reduced when the native A-chain is replaced with the INSL5 A-chain, forming the chimeric peptide R3/I5 [18]. Thus, the relaxin-3 A-chain provides structural stability to the two-chain peptide and the important residues involved in binding and activation of RXFP3 reside exclusively in the B-chain [18,19]. Residues Arg8, Arg12, Ile15, Arg16, Ile19 and Phe20 on the solvent-exposed side of the helical relaxin-3 B-chain have been identified to be important for binding to RXFP3 whereas Arg26 and Trp27 are critical for activation of the receptor [20]. Replacing the last five amino acid residues of the relaxin-3 B-chain C-terminus with a single non-native Arg results in a high affinity antagonist, R3(BΔ23-27)R/I5 [20]. Both the agonist and antagonist analogues have been used as tools for studying relaxin-3 biology [8,11]. However, chemical synthesis of two-chain peptides is costly and complex, and in vivo studies require central administration into the brain; thus, further improved analogues are needed. 

The first single-chain peptide with selectivity and high affinity for RXFP3 was the antagonist R3 B1-22R, which is based on a truncated B-chain in which two Cys residues have been replaced with Ser residues [21]. The R3 B1-22R antagonist lacks the helical structure of native B-chain in solution [21], and the extra flexibility allows it to bind to RXFP3 in a different fashion to the native peptide [22,23,24,25,26,27]. The full-length B-chain, which is unstructured and a weak agonist, can be improved by reintroducing helical structure through helical stapling with dicarba bonds [9,28,29]. Using ring-closing metathesis (RCM), staples introduced at different positions of the relaxin-3 B-chain and with different lengths result in single-chain peptides with regained helicity and significantly improved affinity and activity at RXFP3, as well as improved proteolytic stability [9,28,29]. While the RCM method can provide compounds with excellent pharmacology, the hydrocarbon stapled analogues are low yielding and have poor solubility. The RCM method also often produces mixtures of cis/trans products. To overcome these limitations investigated whether simpler alternative helical support approaches could be introduced into single chain agonists to improve binding and potency. The alternative stapling methodologies employed in this study included cysteine alkylation using halogen staples through reactions with dichloroacetone (DCA) and α,α’-dibromo-*m*-xylene (DBX) [30,31,32]. Another strategy investigated was the incorporation of helicogenic α-aminoisobutyric acid (Aib) residues [33]. The designed analogues were synthesised, and structural and functional effects of modifications monitored by NMR spectroscopy and cell-based assays, respectively.

## 2. Experimental Section

### 2.1. Materials

All standard amino acids were purchased from GL Biochem (Shanghai, China) and Mimotopes (Mulgrave, VIC, Australia). (*S*)-*N*-Fmoc-2-(4-Pentenyl)glycine-OH (Fmoc-Pg) was purchased from ChemPep (Wellington, FL, United States). Fmoc-Trp(Boc) Tentagel S-PHB resin (0.23 mmol/g) was purchased from Rapp Polymere (Tübingen, BW, Germany). Fmoc-Trp(Boc)-Peg-PS resin (0.23 mmol/g) was purchased from Applied Biosystems (Scoresby, VIC, Australia). LC-MS grade formic acid (OPTIMA) was purchased from Thermo Fisher Scientific (Scoresby, VIC, Australia). (*S*)-*N*-Fmoc-2-(4-pentenyl)alanine-OH (Fmoc-Pa), Tris(2-carboxyethyl)phosphine (TCEP), 3,6-Dioxa-1,8-octane-dithiol (DODT), triisopropylsilane (TIPS), Grubb’s catalyst (2nd generation), 1,3-dichloroacetone (DCA), and α,α’-dibromo-*m*-xylene (DBX) were purchased from Sigma Aldrich (Castle Hill, NSW, Australia). All other solvents and chemicals were purchased from Merck (Bayswater, VIC, Australia) and were of peptide synthesis grade. 

### 2.2. Peptide Synthesis

Peptides were synthesised using Fmoc-chemistry-based solid phase peptide synthesis on a 0.125 mmol scale. Sequence assembly was performed manually or on automated peptide synthesisers (CS Bio 336X (Menlo Park, CA, USA) and Biotage Initiator+ Alstra (Uppsala, Sweden)). Fmoc deprotection was carried out using 20% piperidine in DMF before each amino acid was coupled using 8 eq. amino acid, 8 eq. 1 M DIPEA and 8 eq. 0.5 M HBTU at room temperature for 45 min. Unusual amino acids were coupled using 2 eq. Fmoc-Pa or Fmoc-Pg, 1.8 eq. of HBTU, and 4 eq. of DIPEA for a duration of 3 h at room temperature. Residues following the unusual amino acid were coupled using 8 eq. amino acid, 8 eq. HATU and 12 eq. DIPEA, shaken for 4 h at room temperature. In analogues **3**, **4**, **9** and **10**, which contain consecutive Aib residues, the two residues following the first Aib coupling required 8 eq. PyBOP and 16 eq. DIPEA shaken for 20 h at room temperature for complete coupling. Acetylation of peptide N-termini was carried out using 9.2 mmol acetic anhydride and 2.7 mmol DIPEA in 15 mL DMF (2 × 5 min). 

Upon completion of synthesis, the peptides were cleaved from resin using a mixture of TFA/TIPS/DODT/H_2_O (92.5:2.5:2.5:2.5) for 2 h. TFA was evaporated off under vacuum and the peptides were precipitated using ice-cold diethyl ether. Precipitated peptides were redissolved in 50/50 buffer A (0.05% TFA in H_2_O) and buffer B (90% acetonitrile (ACN) and 0.045% TFA in H_2_O), before lyophilisation. The linear peptides were purified using C18 reversed phase columns on a Prominence HPLC system (Shimadzu, Rydalmere, NSW, Australia) with a gradient of buffer A and buffer B. Characterisation of all analogues were conducted using electro-spray ionisation mass spectrometry (ESI-MS) on an API2000 (AB Sciex, Mt Waverly, VIC, Australia). Analogues were analysed for purity using an analytical RP-HPLC C18 column with a flow rate of 1 mL/min or 0.3 mL/min at a 1% gradient, and confirmed as >95% pure.

### 2.3. Hydrocarbon and Halogen Stapling

**Scheme 1:**
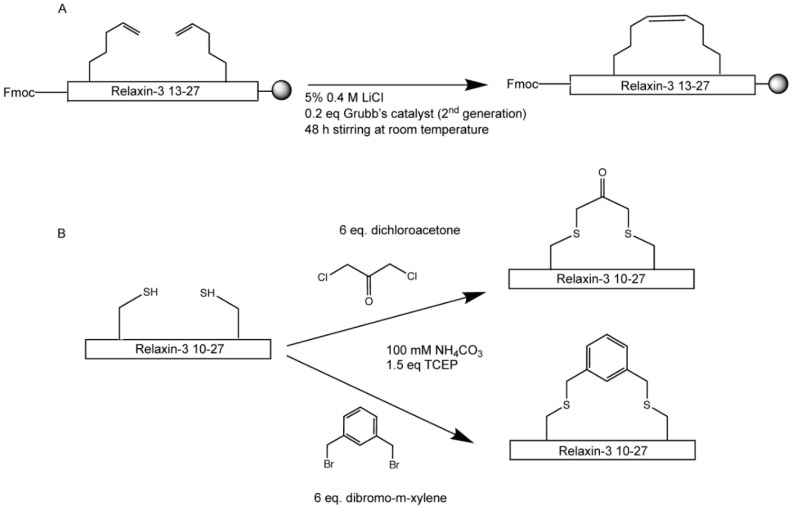
Stabilisation strategies for single-chain relaxin-3 agonists. (**A**) stapling using dicarba strategies. (**B**) stapling using thioether strategies.

Analogue **12** containing Pa residues was stapled using ring-closing metathesis (RCM) on resin, similar to a previously described protocol [34]. The RCM reaction was initiated after the second Pa residue was coupled onto position 13 of relaxin-3 B-chain, and is conducted as shown in Scheme 1A. Briefly, 5% 0.4 M LiCl in DMF and 0.2 eq. 2nd generation Grubb’s catalyst in DCM were added to 0.02 mmol Fmoc-protected on-resin peptide. All mixtures used were degassed before and after addition to the resin. The reaction mixture was shaken in the dark at room temperature for 48 h. Once the hydrocarbon stapling was completed, the protected on-resin peptide was washed with DCM and DMF before continuation of Fmoc deprotection followed by SPPS synthesis.

Analogues **14**–**17** were synthesised and purified using RP-HPLC before thioether stapling was conducted in solution as shown in Scheme 1B. 1.5 eq. TCEP was added to analogues containing minimal amount of AB buffer dissolved in 100 mM NH_4_HCO_3_ (3 mg/mL), and the reaction was stirred for 1 h at room temperature. 6 eq. of DCA or DBX dissolved in minimal DMF was then added to the reaction and further stirred for 2 h at room temperature. The thioether stapling reaction was quenched using 4% TFA and samples diluted with buffer A before purification using RP-HPLC.

### 2.4. NMR Analysis

NMR samples containing 500 μg peptide in 500 μL 90% H_2_O/10% D_2_O (*v*/*v*) at pH 3.5 was prepared. Two-dimensional ^1^H homonuclear total correlation spectroscopy (TOCSY) and nuclear Overhauser effect spectroscopy (NOESY) datasets were recorded with mixing times of 80 ms and 200 ms, respectively, on a 600 MHz Avance III spectrometer equipped with a cryoprobe (Bruker). The program CARA [35] was used to analyse the 2D data after processing using Topspin. Spectra were referenced to 4,4-dimethyl-4-silapentane-1-sulfonic acid at 0.0 ppm, and the secondary shifts were determined using available random coil shifts [36]. 

### 2.5. Competition Binding Assay

Determination of binding affinity in this study was carried out as previously described [37]. Briefly, CHO-K1 cells stably expressing RXFP3 [38] were treated with 5 nM Eu-labelled R3 B1-22R with increasing concentrations of novel analogues. Fluorescence measurement were recorded with 340 nm excitation and 614 nm emission wavelengths. Assays were conducted in triplicates and independently repeated at least three times. Data are presented as mean ± SEM. pK_i_ values were determined using one-site fit Ki with a Kd value of 28 nM. Statistical analyses were conducted using 1-way ANOVA with uncorrected Fisher’s least square difference in GraphPad Prism 8.0.

### 2.6. cAMP Activity Assay

CHO-KI cells stably expressing RXFP3 were used to study the ability of the novel analogues to inhibit forskolin-induced cAMP accumulation. Assays were conducted as previously described [39]. Briefly, 5 μM forskolin was used to stimulate the cells and increasing concentrations of the novel analogues were added to measure the dose-dependent reduction in the cAMP response via activation of the G_i/o_-coupled RXFP3. Each analogue was tested in triplicates and each experiment was independently repeated three times. Data are presented as mean ± SEM and were analysed using GraphPad Prism 8.0. Statistical analyses were conducted using 1-way ANOVA and uncorrected Fisher’s least square difference.

## 3. Results

### 3.1. Peptide Design Rationale and Synthesis

The design of the set of peptides was-based around the success of the hydrocarbon stapling positions of Ac-R3B10-27 [13/17 HC]; Glu13 and Ala17 (Table 1) [28]. First, Aib residues were introduced at the same positions of the relaxin-3 B-chain (analogue **1**), to observe if this simpler strategy is sufficient to induce α-helical structure, improve affinity and activity of single-chain relaxin-3 agonist. Aib residues introduce the same steric constraint on the backbone as the pentenylalanine residues used for hydrocarbon stapling, but avoids the need for an RCM reaction to form a side chain crosslink. Additional Aib residues were also introduced at positions Phe14, Val18, Thr21 or Ser22, none of which have been described as important for relaxin-3/RXFP3 interactions, and indeed previously having been used as stapling positions without adversely affecting ligand-receptor interactions [9,29]. These Aib residues were added into analogues containing Aib at Glu13 and Ala17 to form a series of triple and quadruple substituted agonist peptides, analogues **2**–**10**. We further created analogues of Ac-R3B10-27 [13/17 HC] to understand why this stapling is so favoured and to see if it can be further improved. Analogue **11** is the linear variant of Ac-R3B10-27 [13/17 HC]. It has pentenyl-alanine residues that have not been crosslinked at positions 13 and 17 to interrogate if the addition of hydrophobic side chains, not just the cross link, contribute to binding. Ac-R3B10-27 [13/17 HC] does have solubility issues due to its hydrophobicity, and here four Lys residues creating a solubilisation-tag were added to the N-terminus of the peptide, resulting in analogue **12**, to increase hydrophilicity. We also wanted to investigate how much the additions of steric constraints in the backbone contributes to the helical conformation, so an analogue containing hydrocarbon staple using a Pg staple (**13**) was designed. This variant contains the same side-chain staple as analogue Ac-R3B10-27 [13/17 HC], but lacks the extra steric restriction of the backbone. Despite multiple attempts using different stapling conditions, this analogue could however not be stapled and only its linear version (**13**) could be assessed. Another stapling strategy involving cysteine-based alkylation was also investigated. In this strategy, peptides containing Cys substitutions at Glu13 and Ala17 in the single-chain agonist were stapled using DCA (**14** and **15**) or DBX (**16** and **17**). Acetylated and non-acetylated variants were synthesised to probe the role of the N-terminus. Table 1 lists the analogues containing Aib and side-chain staples. All peptides were synthesised using solid phase peptide synthesis and purified by RP-HPLC to obtain peptides with high degree of purity. HPLC and MS data for all analogues are included as Supplementary Materials.

### 3.2. Helicogenic Amino Acid and Thiol-Based Staples Show Small Improvements in Secondary Structure of Single-Chain Relaxin-3 Agonists 

**Figure 2:**
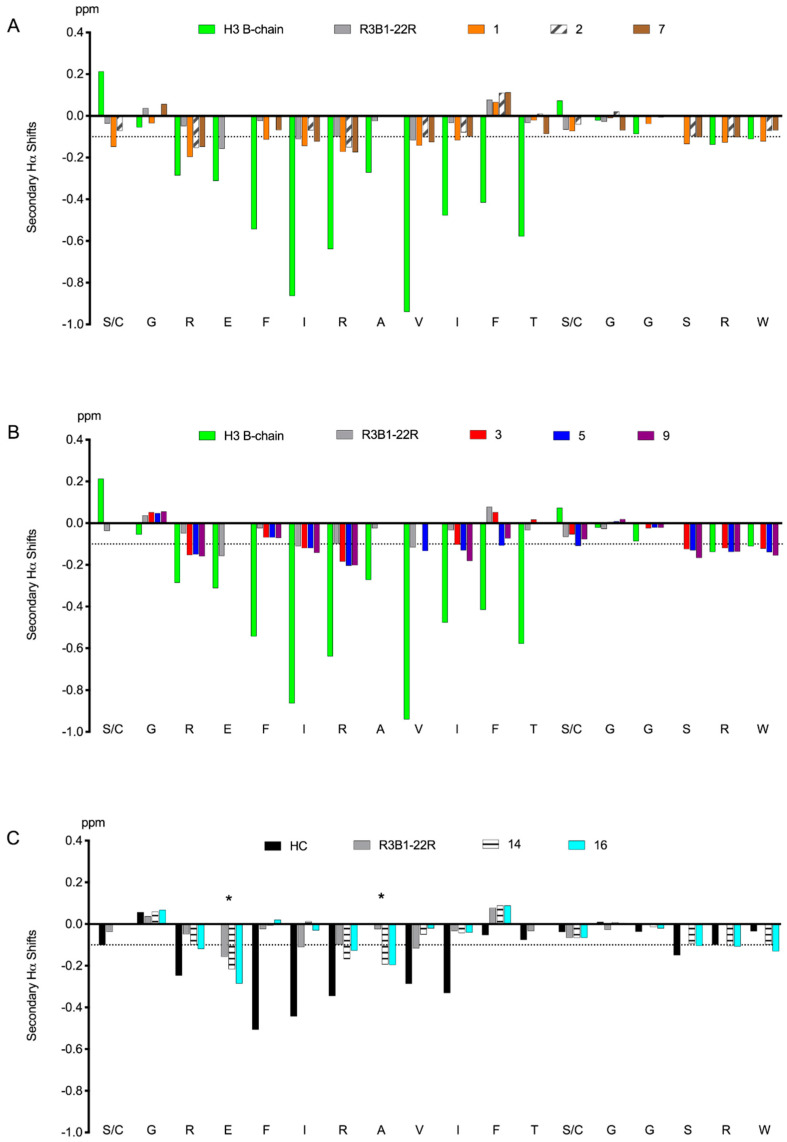
Comparison of secondary Hα chemical shift of Aib containing and stapled relaxin-3 analogues. Secondary Hα chemical shifts are presented as the recorded chemical shift subtracted by the random coil chemical shift for each amino acid. An extended region of negative values indicates an α-helical structure. Analogues were compared to H3 relaxin B-chain of the native peptide or Ac-R3B10-27 [13/17 HC] (HC) and R3B1-22R. (**A**) Comparison between **1** (Ac-R3 B10-27 13, 17 Aib), **2** (Ac-R3 B10-27 13, 14, 17 Aib) and **7** (R3 B10-27 13, 17, 22 Aib), whereas (**B**) shows comparison of **3** (R3 B10-27 13, 17, 18 Aib), **5** (R3B 10-27 13, 17, 21 Aib) and **9** (R3 B10-27 13, 17, 18, 21 Aib). Cysteine-based stapled analogues **14** (R3 B10-27 13, 17 DCA) and **16** (R3 B10-27 13, 17 DBX) are compared in (**C**). * thiol stapling positions. Random coil shifts for Aib and Pa are not available. Oxidised Cys random coil value was for calculation of secondary Hα chemical shift at stapled sites of analogues **14** and **16**.

Helical properties of relaxin-3 agonist analogues containing strategic substitution of native relaxin-3 residues for Aib and alternative cysteine-based stapling were investigated using NMR spectroscopy. Analogues **1, 2, 3, 5**, **7**, **9**, **14** and **16** were studied by NMR spectroscopy and the secondary Hα shifts were determined. The NMR data were of good quality with sharp peaks, but generally had poor signal dispersion. The secondary shifts of these analogues were compared to native H3 relaxin B-chain, the stapled variant Ac-R3B10-27 [13/17 HC], and the single-chain relaxin-3 antagonist, R3 B1-22R. Overall, the Aib containing relaxin-3 analogues showed a slight increase in helical content compared to R3 B1-22R, although the degree of helicity is significantly less than that observed in the native relaxin-3 B-chain (Figure 2, panels A–B). In particular, residues N-terminal to the introduced Aib residues showed more negative secondary shifts compared to R3 B1-22R, which support increased helicity around the modifications, but the effect was local. As illustrated in Figure 2C, the secondary Hα shifts of DCA (analogue **14**) and DBX (analogue **16**) stapled relaxin-3 agonists closely resemble each other. The secondary Hα shifts were more negative at the stapled sites of Cys13 and Cys17 and the residue immediately before them, but other chemical shifts were more similar to R3 B1-22R. Overall, none of the modifications were able to recapitulate the strong helical effect of the hydrocarbon staple of Ac-R3B10-27 [13/17 HC], but the C-terminal tail showed closely matching chemical shifts to both this stapled variant and native relaxin-3.

### 3.3. Aib Residues, but not Cysteine Staples, Can Improve Potency at RXFP3

**Figure 3:**
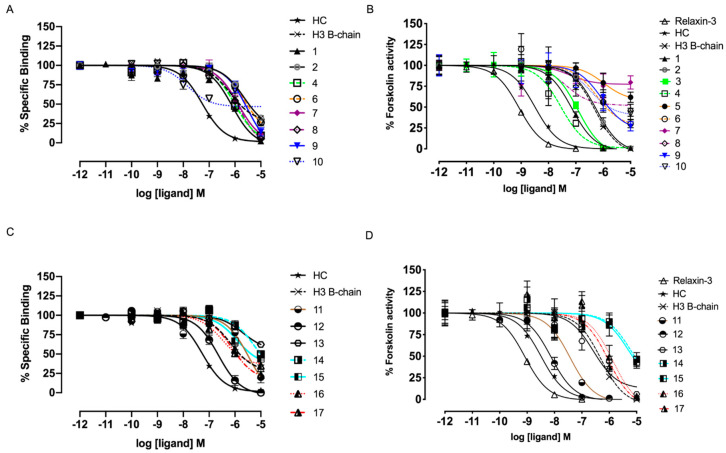
Binding affinity and potency of relaxin-3 agonist analogues at RXFP3. (**A**,**C**) illustrate the binding of the Aib and stapled analogues, respectively. (**B**,**D**) show the potency of Aib containing and stapled analogues, respectively. Data are shown as mean ± SEM of triplicate points from a minimum of three independent experiments. HC represents Ac-R3B10-27 [13/17 HC].

Improving helical structure can improve the interaction with RXFP3 [28]; hence, we investigated whether the modest structural changes in our novel analogues resulted in any gain in binding and potency at RXFP3. To determine their binding affinity, the ability of increasing concentration of analogues to compete with europium labelled R3 B1-22R were measured using CHO cells stably expressing RXFP3 [37]. Their potency at RXFP3 was measured by inhibition of cAMP accumulation induced by forskolin, as RXFP3 couples to an inhibitory G-protein. The binding and activation data are summarised in Table 2 and shown in Figure 3. 

The hydrocarbon stapled relaxin-3 agonist, Ac-R3B10-27 [13/17 HC], was shown to have high affinity (pK_i_ = 7.38) and potency (pEC_50_ = 8.48) in a previous study [28]. First, we looked at the effect of substitutions with Aib residues, restraining the backbone without stapling (Table 2, Figure 3). Analogue **1**, containing Aib residues at the same position as the stapled Ac-R3B10-27 [13/17 HC], did show improvement in binding (pK_i_ = 6.25) and activity (pEC_50_ = 7.17) at RXFP3 compared to the relaxin-3 B-chain, R3B1-27 (pK_i_ = 5.91, pEC_50_ = 5.93), highlighting that the conformational restriction of the backbone is favourable even in the absence of a side chain staple. Addition of further Aib residues had a mixed result. Aib substitution of Val18 (analogue **4**) further improved potency (pEC_50_ = 7.48) compared to the relaxin-3 B-chain. However, Aib substitutions at positions Phe14 (analogue **2**), Thr21 (analogue **6**) or Ser22 (analogue **8**) were found to be unfavoured, which was reflected in both triple and quadruple substituted variants. Variants with Aib at position 22 (analogues **7** and **8**) were poor agonists and only showed a maximum cAMP accumulation inhibition of ~50% at the highest concentration tested (Figure 3). Acetylation of the N-terminus did not largely influence receptor activity.

Second, we tested variants with alternative staples (Table 2, Figure 3). The linear Ac-R3B10-27 [13/17 HC] (**11**) showed similar affinity and activity for RXFP3 as **1** with a pK_i_ of 6.03 and pEC_50_ of 7.38. Thus, the presence of the larger hydrophobic pentenyl-alanine side chains does not improve activity in a linear variant. Additional Lys residues at the N-terminus of R3B10-27 [13/17 HC] were tolerated, with no significant effect on binding or activity of the hydrocarbon stapled analogue **12**. The absence of the methyl group at the Cα in the linear agonist containing Pg residues (analogue **13**) resulted in a substantial loss in affinity for RXFP3, with pK_i_ < 5, consistent with the results from the Aib variant (analogue **1**) and the stapled and linear Pa variants. Alternative thiol-based staples using Cys at positions 13 and 17 of the single-chain relaxin-3 agonist together with DCA and DBX staples were not favourable and did not improve binding or activity of analogues **14**–**17** over R3B1-27.

## 4. Discussion

RXFP3 is pursued as an attractive drug target, as its activation by relaxin-3 controls food intake, stress, arousal and reward-seeking behaviour in animal models [13]. Due to the poor selectivity and high cost of native relaxin-3 production, SAR studies and analogue design have been conducted to develop pharmaceutical drug leads. Recently, a single-chain relaxin-3 agonist, Ac-R3B10-27 [13/17 HC], was designed, synthesised and shown to have improved selectivity for RXFP3. Its helical secondary structure was found to mimic native relaxin-3, as a result of side chain cyclisation using a dicarba bond. In the current study, we investigated whether similar effects can be observed incorporating alternative helical promoting features in the relaxin-3 B-chain. The presence of multiple Aib residues are known to improve peptide helical conformation and stability [33,40]. Hence, a series of analogues containing Aib residues judiciously incorporated at non-essential positions of the relaxin-3 B-chain were designed and compared to Ac-R3B10-27 [13/17 HC]. Furthermore, the versatile strategy of cysteine-based alkylation to create staples with different linker size and functionalities was explored [31,41].

The structure of designed analogues was analysed using solution NMR spectroscopy. Based on the secondary shifts obtained through the NMR studies, we observed that analogue **1**, containing Aib residues replacing Glu13 and Ala17 of the relaxin-3 B-chain, did have increased helical tendencies relative to the linear B-chain antagonist, but the improvement was only minor compared to the stapled peptide Ac-R3B10-27 [13/17 HC]. Increasing the number of Aib substitution at positions that are not known to be involved in the agonist binding to RXFP3 did not markedly further promote a helical nature of the agonists. Thus, the conformational restraints afforded on the backbone by the Aib residues may have some local effects but are not sufficient to nucleate an extended helical conformation. 

The changes in affinity and potency of analogues with Aib residues largely reflect the degree of helicity obtained. The reinstatement of some helical secondary structure in analogue **1** showed improvement in both affinity and activity compared to R3B1-27, but this is weaker in comparison to Ac-R3B10-27 [13/17 HC], which has a much higher degree of helicity. Interestingly, replacement of additional residues with Aib had a mixed result. Analogue **2** also showed a small gain in helicity compared to R3B1-22R, but both its affinity and potency at RXFP3 are more comparable to R3B1-27, which suggests that the Phe14 side chain may contribute to the RXFP3 interaction. Phe14 in native relaxin is buried in the two-chain peptide core; however, we have shown for the linear antagonist that the binding mode of these smaller analogues can be different, thus Phe14 might provide contacts in this context [27]. In contrast, analogue **4** that also contains an Aib at position Val18, was shown to gain significant potency at RXFP3 compared to the relaxin-3 B-chain alone, suggesting this site is amenable for substitutions promoting RXFP3 activity. Increasing the number of Aib substitution further with the addition of Aib at Thr21, again caused a significant reduction in binding and potency as seen in analogues **9** and **10**. A similar drop in affinity and potency can be observed in analogues **5** and **6** that contain the Aib at Thr21 but not Val18. These analogues (**3**–**6**, **9**–**10**) have similar propensity towards helical structure, and hence the difference in the binding affinity and activity would again be more likely due to loss in side-chain functional groups. Therefore, the reduction in potency at RXFP3 can be narrowed down to Thr21, which may be participating in binding and activating RXFP3. The reduced affinity and potency of analogues **9** and **10** is consistent with a recent study where relaxin-3 agonist activity was grafted onto a peptide scaffold based on apamin. The scaffold was successful for introducing helicity and activity in agonists, but when Val18 and Thr21 were additionally substituted with Aib the potency gain was lost despite further strengthening of the helical character [42]. It is likely that Thr21 in the grafted analogue was responsible for the reduced binding and activation of RXFP3. It is interesting to note that Thr21 has never been replaced in native relaxin-3, and given that it is highly conserved in mammalian relaxin-3 sequences and positioned close to the critical C-terminus, it cannot be ruled out that it is involved in binding. It is not an absolutely critical residue, although, as an i-i_+7_ stapled variant, with a dicarba linker between positions 14 and 21 and additional Cα methyl groups, it has been shown to have potency similar to Ac-R3B10-27 [13/17 HC] [9]. Again, this may reflect different binding modes of differently modified peptides. In the single chain antagonist Thr21, replacement with Aib is well-tolerated [27]. Finally, variants containing an Aib residue at position 22 (analogues **8** and **9**) also showed impaired activation. Position 22 in native relaxin-2 comprise a cysteine that is linked to the A-chain. The significance of this position in single chain analogues has been poorly explored, but Ser is favoured over Ala in the antagonist variants [21]. Restricting the backbone using an Aib here may not allow the C-terminal to adopt the correct conformation for receptor activation but it could potentially be amenable to alternative modifications creating new receptor interactions and optimising agonist activity. 

**Figure 4:**
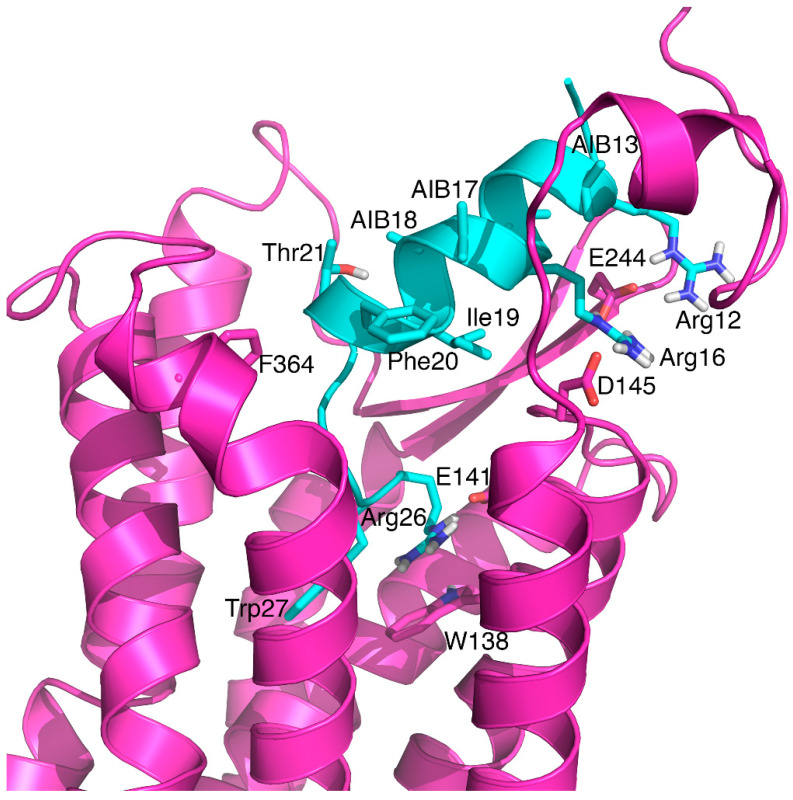
Model of analogue **4** bound to RXFP3. The predicted helical structure allows the same key interactions between arginine residues in relaxin-3 and acidic residues in RXFP3, as well as hydrophobic contacts [22]. Residues in analogue **4** are labelled with three-letter amino acid codes and residue numbers based on the relaxin-3 sequence, and residues in RXFP3 are labelled with single letter amino acid codes and residue numbers.

Creating a precise molecular model of how these analogues interact with RXFP3 is challenging for several reasons. No crystal structure is available of RXFP3 and as shown here the structure of the ligand is still uncertain given its high degree of flexibility even when Aib residues are introduced. Nonetheless, we used a recent model of the complex between relaxin-3 and RXFP3 generated based on homology modelling, the NMR structure of relaxin-3 and extensive mutational data [22] to illustrate how analogue **4** might engage RXFP3. As shown in Figure 4, if a similar binding mode is used the introduced Aib residues are largely on the opposite face of the B-chain helix and not in contact with RXFP3, consistent with only a structural role. Thr21 could potentially form contact with residues in ECL3 or TM7, similar to what has been predicted for Phe20 [22].

Cysteine-based stapling is advantageous for synthesis, as it does not require unnatural amino acids and stapling can be done in solution after purification. The alternative staples at positions Glu13 and Ala17 in analogues **14** and **16**, however, had limited effect in terms of promoting α-helical structure as shown by the NMR studies, with the variants still remaining primarily random coil. This is in stark contrast to the significant helical induction previously reported for Ac-R3B10-27 [13/17 HC], for which a fully helical structure could be determined on the basis of a large number of medium range NOEs [28]. The Pa dicarba staple in this variant contains eight atoms, whereas the use of DCA and DBX cyclised to Cys, resulted in seven-atom and nine-atom staples, respectively. This may suggest that an eight-atom linker is optimal for relaxin-3 stapling; however lactam bond stapling resulting in an eight-atom linker has previously be shown not to be effective in rescuing structure or activity [28]. Studies have also demonstrated that nine-atom staples using DBX conjugated to Cys or DCA to homocysteine conveys an optimal linker length to stabilise one turn of α-helix at *i*,*i*+4 position in other peptides [30,31]. The lack of secondary structure in **14** and **16** compared to Ac-R3B10-27 [13/17 HC] is more likely to be a result of the lack of the additional Cα methyl groups. The benefit of these is evident from their positive influence on both structure and activity of analogue **1**. To further confirm this, we attempted to make a stapled variant using Pg residues, creating an identical side chain cross link without the Cα substitutions. The cross linking of this variant failed despite multiple attempts, perhaps an indication that the Cα methyl groups of Pa order the backbone in a conformation allowing the side chain cyclisation to take place. We conclude the effectiveness of different types of staples in promoting helicity is likely to be highly sequence specific and for relaxin-3 the extra Cα methyl groups are highly favoured and it is possible that combining these with other types of side chain linkages may evoke an improvement in helicity similar to Ac-R3B10-27 [13/17 HC].

Given the thiol staples used here did not significantly improve structure of the relaxin-3 analogues, it was not surprising to see that they had no effect on binding and activation of RXFP3. Relaxin-3 agonists, relative to antagonists, require a more structured conformation with the correct spatial positioning of different features for efficient interaction with, and activation of, the receptor [22,28]. The nature of the staple used can influence activity in cases where it is not just a structural support but contacts the receptor. The degree of hydrophobicity sometimes correlates more with activity than helicity of a peptide, in particular if cellular permeability is required [43,44]. The staple position used here would not be expected to contribute to receptor interactions, given its position in native relaxin-3. As pointed out, changes in binding mode can, however, not be ruled out for these smaller peptides. The analogues designed here contain staples with different degree of hydrophobicity, *m*-xylene and dichloroacetone. Hydrophilic lactam stapling on relaxin-3 agonists and antagonists have previously been shown not to improve binding affinity for RXFP3 [27,39] unlike the hydrocarbon staple [28], which is more hydrophobic in nature [45]. Importantly, binding and affinity comparison between the Aib substituted and Pa hydrocarbon-containing, but not cross linked, analogues indicate that hydrophobicity may not be playing a major role at these sites since **1** and **11** have similar affinity for RXFP3 even though the latter is significantly more hydrophobic than the former. 

In this study, we made acetylated and non-acetylated versions of our analogues. Although N-terminal acetylation can alter peptide function [46], in the case of relaxin-3, the difference in binding and potency of acetylated and non-acetylated analogues were not markedly different. No obvious differences in secondary structure where noted when the N-terminus was acetylated; however, to avoid unfavourable electrostatic interactions between a positively charged N-terminus and the helix dipole, acetylation of these shorter analogues makes sense and is likely to also provide better in vivo stability. The Ac-R3B10-27 [13/17 HC] lead molecule is poorly soluble because of its hydrophobicity. We also made a variant of this with an additional solubilising tag comprising four Lys residues. This was well tolerated confirming the N-terminus is a good position to add features in order to change physicochemical properties or in vivo distribution. In particular, modifications are likely to be required for these peptides to be able to pass the blood-brain barrier (BBB) and reach the targeted RXPF3-rich sites. Several peptide sequences able to promote BBB penetration have been identified or developed recently, including from both naturally occurring peptides such as apamin and chlorotoxin [47,48,49] and from library screening approaches [50,51,52]. Such sequences have been shown to be effective in bringing even large cargo such as nano- and microparticles across the BBB, and thus are likely to also be effective in promoting translocation of relaxin-3 analogues [53]. The N-terminal region of single chain relaxin agonists appears ideal for conjugation of such sequences to further improve their potential.

## 5. Conclusions

Novel relaxin-3 analogues containing alternative helical stabilising residues and staples were successfully synthesised and characterised. While the simpler and more cost-effective strategies investigated failed to pharmacologically improve on the existing design of a single-chain relaxin-3 agonist, we show that Aib substitution at the same positions as the dicarba linked analogue does improve helicity, and by incorporating a third Aib at position Val18 significant potency gains relative to the linear B-chain can be achieved. Positioning of Aib at other positions and cysteine-based stapling were not favoured from a structural or functional perspective. The use of Cα methyl groups when considering stapling strategies appears to be critical. Our study furthermore highlights contributions of side chains to receptor interactions, most notably from Thr21, which may be an overlooked feature of native relaxin-3. Further design can build on these data, also considering the need for an ideal agonist to be able to cross the BBB for in vivo studies of the signalling system, as RXFP3 is predominantly expressed in the brain.

## Figures and Tables

**Table 1:** Peptide sequences of novel relaxin-3 agonist analogues.

Ligand	Sequence	Analogue
H3 B-chain	RAAPYGVRLSGREFIRAVIFTSGGSRW	
R3B 1-22R ^C^	RAAPYGVRLSGREFIRAVIFTSR	
Ac-R3 B10-27 13,17 Pa ^b^	Ac-SGR(**Pa**)FIR(**Pa**)VIFTSGGSRW	**Ac-R3B10-27 [13/17 HC]**
Aib variants		
Ac-R3 B10-27 13,17 Aib	Ac-SGR(**Aib**)FIR(**Aib**)VIFTSGGSRW	**1**
Ac-R3 B10-27 13,14,17 Aib	Ac-SGR(**Aib**)(**Aib**)IR(**Aib**)VIFTSGGSRW	**2**
R3 B10-27 13,17,18 Aib	SGR(**Aib**)FIR(**Aib**)(**Aib**)IFTSGGSRW	**3**
Ac-R3 B10-27 13,17,18 Aib	Ac-SGR(**Aib**)FIR(**Aib**)(**Aib**)IFTSGGSRW	**4**
R3 B10-27 13,17,21 Aib	SGR(**Aib**)FIR(**Aib**)VIF(**Aib**)SGGSRW	**5**
Ac-R3 B10-27 13,17,21 Aib	Ac-SGR(**Aib**)FIR(**Aib**)VIF(**Aib**)SGGSRW	**6**
R3 B10-27 13,17,22 Aib	SGR(**Aib**)FIR(**Aib**)VIFT(**Aib**)GGSRW	**7**
AC-R3 B10-27 13,17,22 Aib	Ac-SGR(**Aib**)FIR(**Aib**)VIFT(**Aib**)GGSRW	**8**
R3 B10-27 13,17,18,21 Aib	SGR(**Aib**)FIR(**Aib**)(**Aib**)IF(**Aib**)SGGSRW	**9**
AC-R3 B10-27 13,17,18,21 Aib	Ac-SGR(**Aib**)FIR(**Aib**)(**Aib**)IF(**Aib**)SGGSRW	**10**
Hydrocarbon variants		
Ac-R3 B10-27 13,17 Pa ^a^	Ac-SGR(**Pa**)FIR(**Pa**)VIFTSGGSRW	**11**
4K-R3 B10-27 13,17 Pa ^b^	KKKKSGR(**Pa**)FIR(**Pa**)VIFTSGGSRW	**12**
Ac-R3 B10-27 13,17 Pg ^a^	Ac-SGR(**Pg**)FIR(**Pg**)VIFTSGGSRW	**13**
R3 B10-27 13,17 DCA ^b^	SGR**C**FIR**C**VIFTSGGSRW	**14**
AC-R3 B10-27 13,17 DCA ^b^	Ac-SGR**C**FIR**C**VIFTSGGSRW	**15**
R3 B10-27 13,17 DBX ^b^	SGR**C**FIR**C**VIFTSGGSRW	**16**
AC-R3 B10-27 13,17 DBX ^b^	Ac-SGR**C**FIR**C**VIFTSGGSRW	**17**

The top three variants have been previously reported and were used as reference compounds. Pa; (S)-2-(4-pentenyl) alanine). Pg; (S)-2-(4-pentenyl) glycine). DCA; dichloroacetone. DBX; α,α’- dibromo-*m*-xylene. ^a^; linear analogue. ^b^; stapled analogue. All analogues were synthesised with an acidic C-terminus. The antagonist ^C^ has an amidated C-terminus.

**Table 2:** Effect of helical promoting residue and stapling introduction on affinity and potency at RXFP3.

Peptide	Analogue	pK_i_ [logM] ^c^	pEC_50_ [logM]
R3B1-27	**H3 B-chain**	5.91 ± 0.21 ^d^	5.93 ± 0.02 ^d,i^
R3 B10-27 13,17 Pa ^b^	**Ac-R3B10-27** **[13/17 HC]**	7.38 ± 0.03	8.48 ± 0.06 ^i^
Aib variants			
Ac-R3 B10-27 13,17 Aib	**1**	6.25 ± 0.12 ^e^	7.17 ± 0.11 ^d,g^
Ac-R3 B10-27 13,14,17 Aib	**2**	5.83 ± 0.18 ^d^	6.26 ± 0.06 ^d^
R3 B10-27 13,17,18 Aib	**3**	ND	7.04 ± 0.04 ^d,g^
Ac-R3 B10-27 13,17,18 Aib	**4**	6.12 ± 0.11 ^d^	7.48 ± 0.20 ^e,f^
R3 B10-27 13,17,21 Aib	**5**	ND	5.99 ^d^
Ac-R3 B10-27 13,17,21 Aib	**6**	5.77 ± 0.28 ^d^	5.73 ± 0.04 ^d^
R3 B10-27 13,17,22 Aib	**7**	5.95 ± 0.05 ^d^	<5
AC-R3 B10-27 13,17,22 Aib	**8**	6.02 ± 0.08 ^d^	<5
R3 B10-27 13,17,18,21 Aib	**9**	5.70 ± 0.19 ^d^	5.75 ± 0.27 ^d^
AC-R3 B10-27 13,17,18,21 Aib	**10**	<5	5.49 ± 0.09 ^d^
Stapled variants			
Ac-R3 B10-27 13,17 Pa ^a^	**11**	6.03 ± 0.45 ^d^	7.38 ± 0.16 ^e,f^
4K-R3 B10-27 13,17 Pa ^b^	**12**	6.74 ± 0.16 ^h^	8.19 ± 0.14 ^f^
Ac-R3 B10-27 13,17 Pg ^a^	**13**	<5	6.51 ± 0.27 ^d^
R3 B10-27 13,17 DCA ^b^	**14**	<5	5.21 ± 0.75 ^d^
AC-R3 B10-27 13,17 DCA ^b^	**15**	5.32 ± 0.21 ^d^	5.32 ± 0.76 ^d^
R3 B10-27 13,17 DBX ^b^	**16**	6.00 ± 0.44 ^d^	5.98 ± 0.07 ^d^
AC-R3 B10-27 13,17 DBX ^b^	**17**	5.98 ± 0.04 ^d^	6.07 ± 0.03 ^d^

Pa; (S)-2-(4-pentenyl) alanine). Pg; (S)-2-(4-pentenyl) glycine). DCA; dichloroacetone. DBX; α,α’- dibromo-*m*-xylene. ^a^; linear analogue. ^b^; stapled analogue. All analogues were synthesised with an acidic C-terminus. ^c^ pK_i_-values are calculated based on the K_d_ 27.9 ± 9.4 nM for Eu-R3 B1-22R. ^d^ *p* < 0.001 and ^e^ *p* < 0.01 compared to Ac-R3B10-27 [13/17 HC]. ^f^ *p* < 0.001, ^g^ *p* < 0.01 and ^h^ *p* < 0.05 compared to R3 B1-27. ^i^, from [28].

## Supplementary Materials

The following are available online at https://www.mdpi.com/2227-9059/8/10/415/s1, Figure S1: Mass spectra of analogues **1**–**6** and their corresponding analytical HPLC traces, Figure S2: Mass spectra of analogues **7**–**12** and their corresponding analytical HPLC traces, Figure S3: Mass spectra of analogues **13**–**17** and their corresponding analytical HPLC traces.
