# Supplementary material for: Exploring the Use of Helicogenic Amino Acids for Optimising Single Chain Relaxin-3 Peptide Agonists
Source: Biomedicines. 2020 Oct 14;8(10):415. doi: 10.3390/biomedicines8100415 (PMC7602263; doi:10.3390/biomedicines8100415)
Supplement: Supplementary file 1 [file biomedicines-08-00415-s001.pdf]

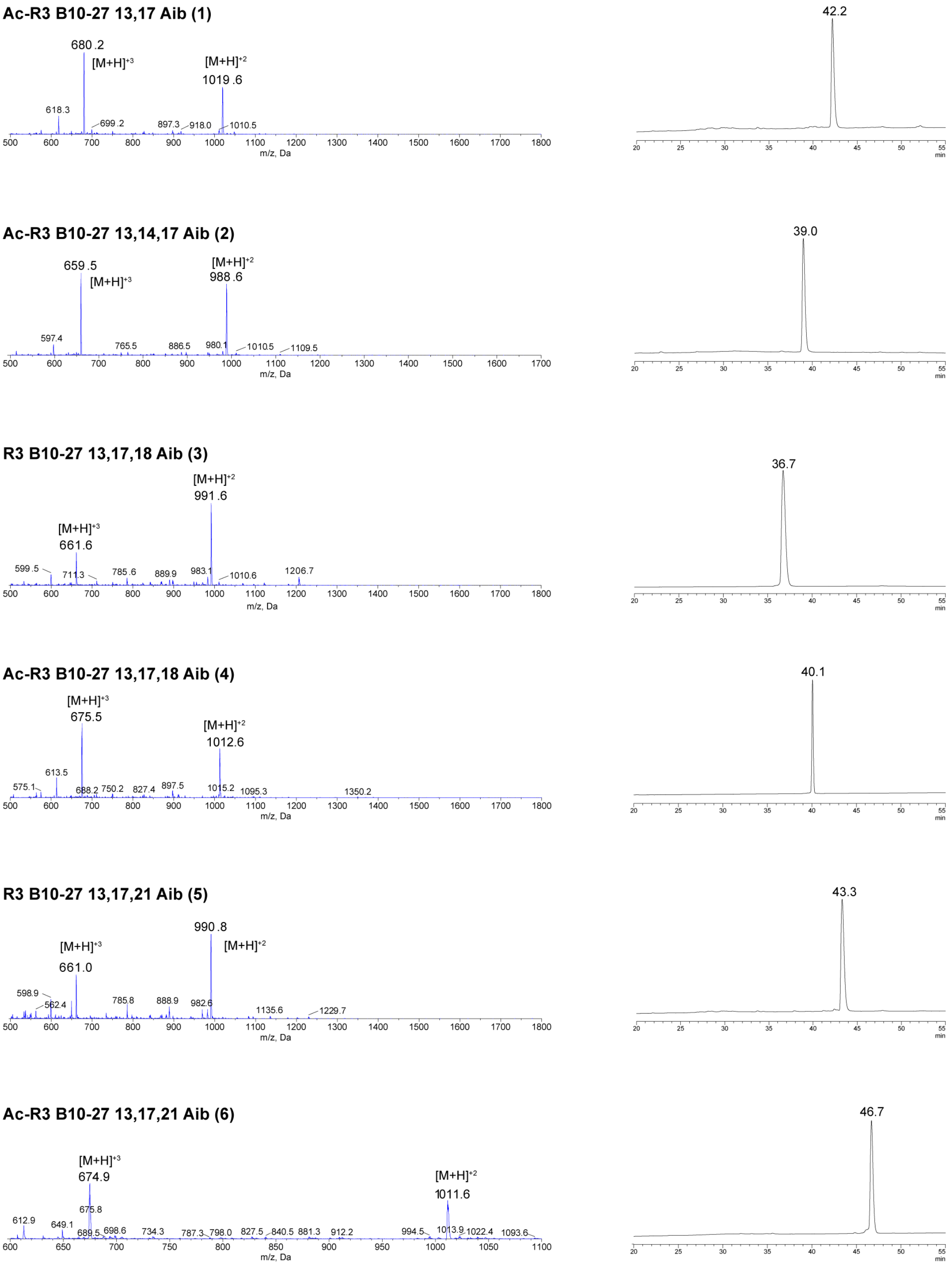

Figure S1. Mass spectra of analogues 1 – 6 and their corresponding peak retention time in analytical HPLC.

R3 B10-27 13,17,22 Aib (7)

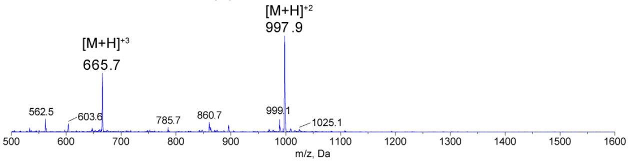

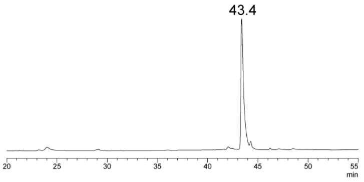

AC-R3 B10-27 13,17,22 Aib (8)

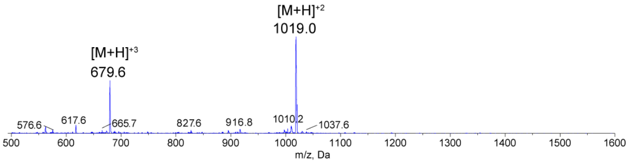

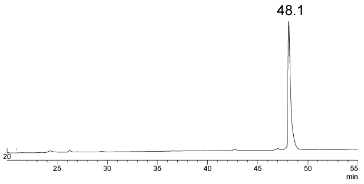

R3 B10-27 13,17,18,21 Aib (9)

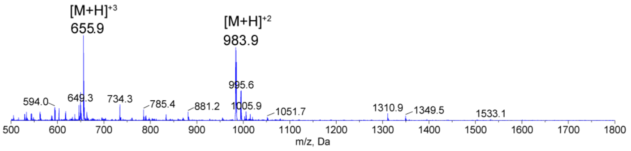

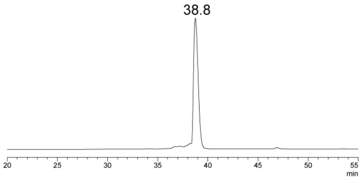

AC-R3 B10-27 13,17,18,21 Aib (10)

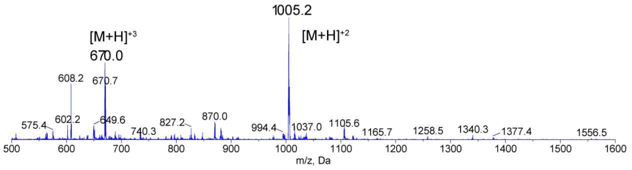

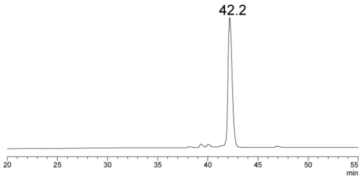

Ac-R3 B10-27 13,17 Pa[a] (11)

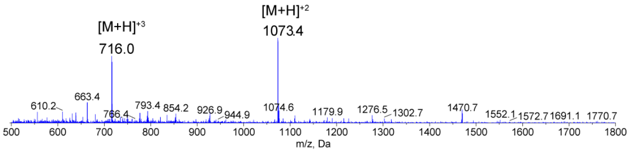

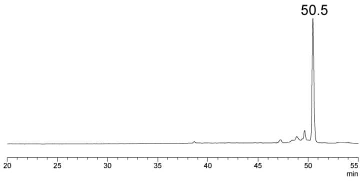

4K-R3 B10-27 13,17 Pa[b] (12)

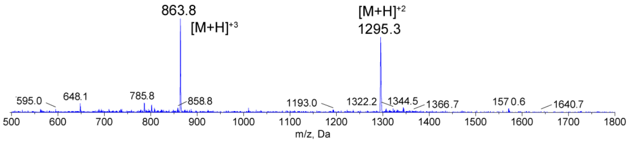

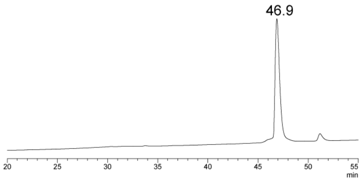

Figure S2. Mass spectra of analogues 7 – 12 and their corresponding peak retention time in analytical HPLC.

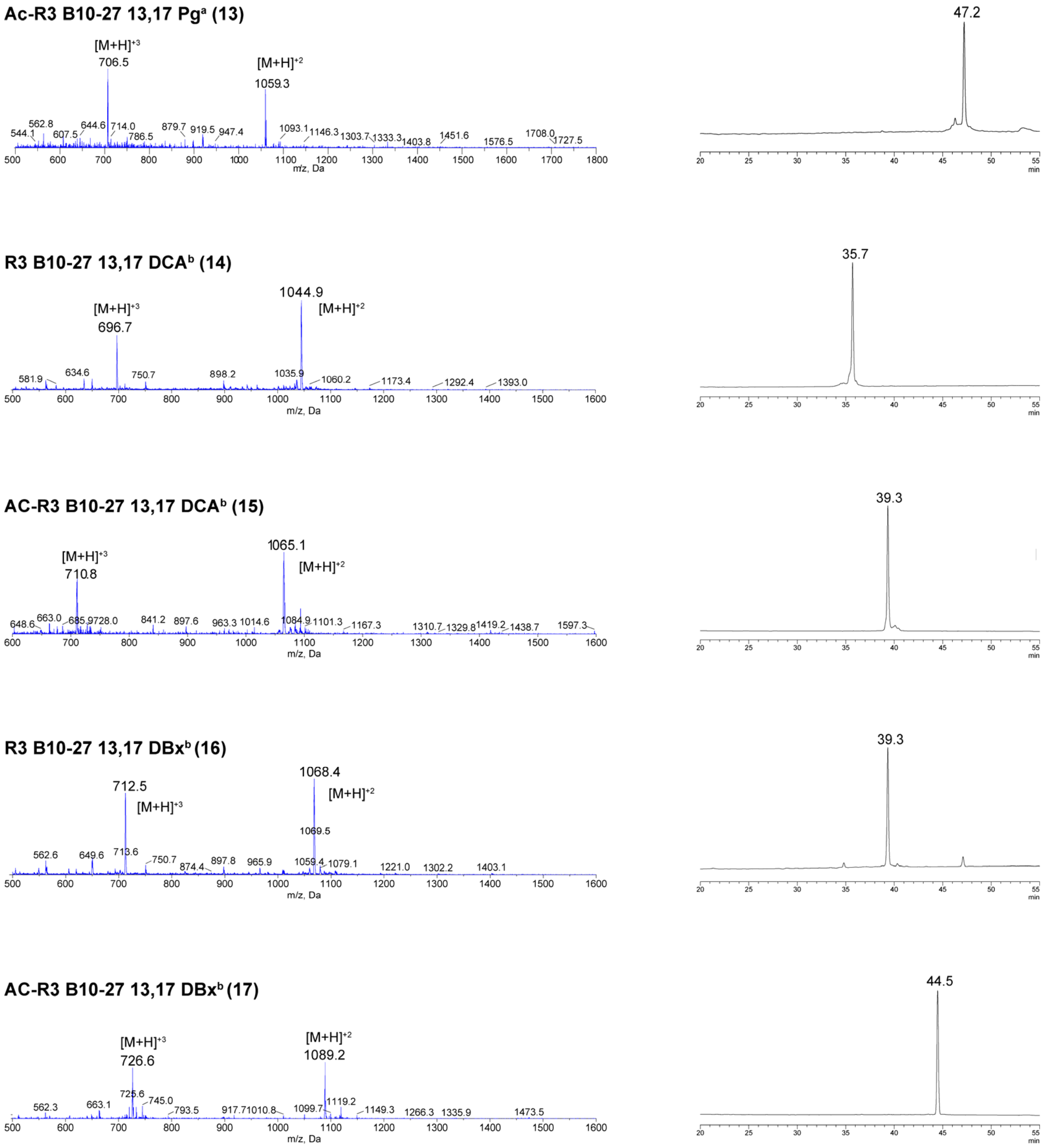

Figure S3. Mass spectra of analogues 13 – 17 and their corresponding peak retention time in analytical HPLC.
